# Harmonisation of imaging dosimetry in clinical practice: practical approaches and guidance from the ESR EuroSafe Imaging initiative

**DOI:** 10.1186/s13244-020-00859-6

**Published:** 2020-03-30

**Authors:** Eliseo Vano, Guy Frija, Wolfram Stiller, Efstathios Efstathopoulos, Claudio Granata, Reinhard Loose, Graciano Paulo, Dean Pekarovic, Johan Sjöberg, Lluís Donoso-Bach, on behalf of the European Society of Radiology (ESR)

**Affiliations:** 1 Radiology department (Medical Physics), Complutense University, Madrid, Spain; 2 Paris Georges Pompidou European Hospital, Paris, France; 3 Diagnostic and Interventional Radiology (DIR), Heidelberg University Hospital, Heidelberg, Germany; 4 2nd Department of Radiology, National and Kapodistrian University of Athens, Athens, Greece; 5 IRCCS Istituto Giannina Gaslini, Genova, Italy; 6 Institute of Medical Physics, Nürnberg, Germany; 7 Instituto Politécnico de Coimbra - Escola Superior de Tecnologia da Saúde de Coimbra (ESTeSC), Coimbra, Portugal; 8 Institute of Radiology, University Medical Centre, Ljubljana, Slovenia; 9 Medical Radiation Physics and Nuclear Medicine, Stockholm, Sweden; 10 Department of Medical Imaging, Hospital Clínic of Barcelona, University of Barcelona, Barcelona, Spain

**Keywords:** Patient exposure, Occupational exposure, Radiation dose, Medical imaging, Regulation, Optimisation

## Abstract

The European Directive 2013/59/EURATOM requires member states of the European Union to ensure justification and optimisation of the radiological procedures and to include information on patient exposure as part of the report of the examinations. The EuroSafe Imaging campaign of the European Society of Radiology created a working group (WG) on “Dosimetry for imaging in clinical practice” with the aim to help with the dosimetry aspects required by European and national regulations. The primary focus topics were selected and a survey among the experts of the WG, allowed suggesting some initial consensus approaches.

For information on patient exposure, it was agreed to include the dosimetric values reported by the imaging modalities (validated by a medical physics expert). It was also suggested to prepare educational material on dosimetric quantities for patients. Individual optimisation was considered a challenge, especially for interventional procedures. In these cases, patient and occupational doses should be part of the global optimisation process and trigger levels should be defined to avoid skin radiation injuries. Diagnostic Reference Levels (DRLs) always need to be considered for comparison with periodic patient dose audits. In the case of accidental or unintended exposures, a report should be produced for the Quality Assurance programme, together with an educational note to avoid the repetition of incidents. Dose registry and management systems should allow fulfilling the regulatory requirements of national and European regulations. In a second step, and after the initial experience with the Directive implementation, a wider survey will be considered.

## Key points

- The 2013/59/Euratom Directive should have been transposed to national legislation and implementation should be harmonised where possible.
- The Dosimetry for imaging in clinical practice WG is working to help with this harmonisation.
- The WG initially covered information on exposures, individual optimisation and proper use of dose registry and management systems.

## Patient summary

The European Directive 2013/59/Euratom sets out the basic safety standards for protection against the dangers arising from exposure to ionising radiation. This directive must be implemented by all radiology departments in Europe and therefore impacts upon all European radiologists. This paper aims to provide support to radiology departments to interpret relevant aspects of the European Directive and suggest pragmatic approaches to implement these into daily working practices.

This directive is also important for patients as it is designed to ensure adequate radiation protection is in place for anyone undergoing medical imaging procedures. All medical radiation procedures will have to be properly justified and optimised (to ensure doses are as low as possible compatible with the required diagnostic information).

The Directive may be complicated to implement in some respects because it only sets out the minimum standards required. It is then up to individual countries or radiology departments to interpret these when implementing. This means that different interpretations and questions have been raised by professionals as well as by scientific and professional societies.

The Dosimetry for imaging in clinical practice working group aims to help European Radiology Departments with the interpretation, implementation and harmonisation of the dosimetry aspects required by the European Directive.

## Introduction

The EuroSafe Imaging campaign is an initiative under the ESR (European Society of Radiology). In 2019, EuroSafe Imaging launched a new Working Group (WG) on “Dosimetry for imaging in clinical practice” with the aim to suggest realistic approaches to fulfil the dosimetric aspects required by the European Directive 2013/59/EURATOM [1, 2] to audit the radiation safety in medical imaging, including the proper use of Diagnostic Reference Levels (DRLs) [3, 4]. One additional and relevant task for the WG was to help in the implementation of the automatic patient dose registries and the appropriate validation methods for the dosimetric results.

The European Directive on Basic Safety Standards was published in January 2014 [1] and European Member States should have completed the transposition into national regulations by February 2018, but a good and harmonised implementation in clinical practice is expected to still need several years.

During these two periods (transposition and implementation of the Directive), several questions and different interpretations have been raised by professionals and by scientific and professional societies.

The ESR is trying to help in the implementation process using the working groups established as part of the EuroSafe Imaging initiative. The different opinions and interpretations of the Directive by the members of the ESR and delegates from other cooperative societies and organisations involved in the WGs, have been taken into account. The “Dosimetry for imaging in clinical practice” WG includes delegates from CIRSE (Cardiovascular and Interventional Radiological Society of Europe), EFOMP (European Federation of Organisations for Medical Physics), EFRS (European Federation of Radiographer Societies) and COCIR (European Coordination Committee of the Radiological Electromedical and Healthcare IT Industry).

Of course, the suggested practical approaches for the implementation of the Directive should always follow the strict fulfilment of the Directive and other existing national regulations. However, it is relevant to consider the practical aspects of clinical imaging and the experience of the professionals involved: Radiologists (and other clinicians using X-rays for diagnostic or interventional purposes), medical physicists, radiographers and industry representatives.

The national competent authorities may also consider the opinions and approaches suggested by the EuroSafe Imaging Working Group during the implementation period, if appropriate.

EuroSafe Imaging presented a second “Call for Action” in 2018 [5] to contribute to achieving the campaign’s aims and to guide all activities developed by EuroSafe Imaging. These actions are supporting the Bonn Call for Action, issued in 2012 by the International Atomic Energy Agency (IAEA) and the World Health Organisation (WHO) [6].

Dosimetry for imaging in clinical practice is important for most of the EuroSafe Imaging Call for Action 2018 actions [5] but it is particularly relevant for:

- Action 2: Develop clinical diagnostic reference levels (DRLs) for adults and children.
- Action 4: Promote dose management systems to establish local, national, and European DRLs.
- Action 6: Implement a clinical audit tool for imaging to improve the quality of patient care.
- Action 11: Establish a dialogue with industry regarding improvement of radiological equipment, the use of up-to-date equipment (e.g. dose management systems) and the harmonisation of exposure indicators.
- Action 12: Improve information for and communication with patients about radiological procedures, related benefits, and possible risks.

It should be noted that in addition to the European regulations, the European Guidelines on Clinical Audit [7, 8] recommend, “the assessment of patient dose from X-ray procedures should be among the necessary physical parts of all clinical audits”.

The aims of this paper are:

- To describe the initial priorities selected by the WG on “Dosimetry for imaging in clinical practice”;
- To summarize the results of a survey collecting the opinions of the experts involved in the WG, and
- To suggest actions helping in the implementation of the new European Directive with the advantages of the dose management systems.

## Material and methods

The initial step was the selection of the main topics to fulfil the objectives of the WG (to help in the dosimetry aspects for implementation of the Directive, and to profit from the automatic patient dose registries) carried out by E-mail to the 11 European experts involved in the EuroSafe Imaging WG (radiologists, medical physicists, radiographers and industry engineers).

The eight topics initially considered as the most relevant to collect the opinions and suggestions were:

1. Information on medical exposure for patients (Art. 58.b of the European directive 2013/59/EURATOM: Member States shall ensure that information relating to patient exposure forms part of the report of the medical radiological procedure).
2. Individual optimisation (Art. 5.b of the European directive: The optimisation of the protection of individuals subject to medical exposure shall apply to the magnitude of individual doses and be consistent with the medical purpose of the exposure).
3. Accidental and unintended exposures (Art. 63.c of the European directive: Member States shall ensure that for all medical exposures the undertaking implements an appropriate system for the record keeping and analysis of events involving or potentially involving accidental or unintended medical exposures, commensurate with the radiological risk posed by the practice).
4. Dosimetric trigger levels (for individual procedures).
5. Comparison with Diagnostic Reference Levels (DRLs).
6. Role of the automatic dose registry and management systems.
7. Dosimetric information for the practitioner.
8. Dosimetric information for the referrer.

The second step was to collect the answers of a questionnaire. The 11 WG experts completed the questionnaire (Appendix 1) and suggested particular comments to some of the offered answer options, when appropriate. Seven experts (out of 11) completed the questionnaire.

Part of the received comments and suggestions have also been considered for the preparation of scientific sessions for the ECR 2020 (learning objectives or potential topics to discuss with the audience).

One aspect included in the survey was the advantages of automatic dose registries introduced in many European hospitals. Most of these registries are for patient doses but in some cases (e.g. interventional practices) also staff doses may be included.

The distributed questionnaire (after the refinements suggested by the experts of the WG) is included in the Annex.

The survey also included the topic on the advantages of simultaneously monitoring and managing patient and occupational exposures for interventional radiology [9].

The registration of patient, and sometimes, occupational exposure, for all the radiation events (as part of the DICOM Radiation Dose Structured Reports –RDSR– in the case of patients) allows new possibilities for the global optimisation during interventional practices. The correlation of occupational and patient exposures is recommended by the ICRP [9]. Information in real time inside the catheterisation rooms, not only on patient exposure, but also on occupational exposure during the procedures [10] is helpful for a global optimisation approach.

Some of the existing problems in reporting occupational exposures from interventionists to international organisations as UNSCEAR (United Nations Scientific Committee on the Effects of Atomic Radiation) may be derived from the difficulty to identify the professionals groups involved in these practices. They could be from different clinical services (Radiology, Cardiology, Vascular Surgery, Urology, Gastroenterology, etc.) and sometimes, the occupational dosimetric data may be reported from a mix of professionals performing fluoroscopy guided procedures including others using more conventional imaging techniques. In these cases, the mean/median occupational dose values may result quite low and it is difficult to identify (by the regulatory authorities or by the radiation protection services) the real sub-group of professionals having a relevant occupational radiation risk.

One way to improve these data collection could be to ask the hospitals, to identify “in origin” the professionals (may be medical doctors, nurses and or technicians) involved in interventional procedures (from all the clinical services) to allow radiation protection services and regulatory authorities to process independently the occupational exposure data from this group of professionals.

## Results

This is the summary of the survey among the experts of the WG:

- Information on patient exposure for patients.
  - **The preferred option for the majority of experts was to inform on “the dose values and units, reported by the X-ray system”.**
  - Other potential options, as informing on the effective doses derived from the imaging procedures or the estimation of the equivalent time in background radiation, were not considered appropriate at this step.
  - Comment: If this approach is selected (“the dose values and units, reported by the X-ray system”) in the future, it will be appropriate to produce educational material for patients informing on the benefit of the procedures and the meaning of these exposure quantities and values reported by the X-ray systems (imaging modalities).
- Individual optimisation.
  - **The majority of experts highlighted the following aspect “Consider patient and staff doses for interventional procedures”.**
  - Other suggested options (supported by some of the experts) were “comparing a group of procedures with Diagnostic Reference Levels (DRLs)” and “consider individual optimisation if individual doses are much higher than DRLs”.
- Accidental and unintended exposures.
  - **All the experts agreed with the following priority “If suspected an accidental or unintended exposure, record and analyse the dose parameters (based on physical quantities) and produce a report for the quality assurance committee”.**
  - Other suggested relevant action (supported by some of the experts) was “To produce an educational note for radiographers, radiologists and medical physicists”.
- Dosimetric trigger levels (for individual procedures).
  - **All the experts agreed with the following priority “Trigger levels should be established for interventional procedures to alert on the risk of potential skin injuries”.**
  - Other suggested relevant action (supported by some of the experts) was “For all imaging procedures, when dosimetric values are much higher than the DRLs”.
- Comparison with Diagnostic Reference Levels (DRLs).
  - **All the experts agreed with the following priority “The comparison with DRLs should be made at least, once per year and after changes in the X-ray unit or in the imaging protocols”.**
  - Other suggested relevant action (supported by some of the experts) was “Continuously if the patient dose registry allow doing it”.
- Role of the dose registry and management systems.
  - **All the experts agreed with the following priority “These systems should allow fulfilling the regulatory requirements (directive 2013/59/EURATOM) on patient dose registration”.**
  - Another suggested relevant aspect (supported by most of the experts) was “These systems should include a validation/correction factor periodically certified by a medical physics expert”.
- Dosimetric information for the practitioner
  - **All the experts agreed that the practitioner should have information on the physical quantities offered by the X-ray system for the different imaging modalities.**
  - Other suggested relevant aspect (supported by some of the experts) was that the practitioner should also have information on the diagnostic reference levels.
- Dosimetric information for the referrer
  - **For this question, there was no agreement between the experts for the options offered (physical dosimetric quantities, effective doses, or diagnostic reference levels). This aspect would need further discussion.**

## Discussion and conclusions

The new European Council Directive 2013/59/Euratom on basic safety standards [1] should already have been transposed to the national legislation for all the member states of the European Union and the implementation should be harmonised as much as possible. The medical and other scientific and professional societies (and the radiology industry) will have a key role on this harmonisation for the benefit of the radiation safety of patients and radiology professionals.

The EuroSafe Imaging WG on “Dosimetry for imaging in clinical practice” is working to help on this harmonisation, looking for the best interpretation of certain articles of the directive and suggesting some pragmatic approaches for the dosimetric aspects in the daily work.

From the topics initially selected by the WG, and for the one concerning the information on patient exposure, it has been suggested that the easiest option will be to use the exposure values reported by the X-systems (validated periodically by a medical physics expert). The WG also proposed to prepare educational material for patients, on the dosimetric quantities.

Individual optimisation is also a challenge, especially for high-dose procedures (e.g. some interventional procedures), and exposure of staff should be considered together with patient exposures. DRLs need to be taken into account (with periodic patient dose audits) and trigger levels should be established to avoid skin radiation injuries [11]. In the case of accidental or unintended exposures, a report should be produced for the Quality Assurance programme together with an educational note to avoid the repetition of the incidents.

The dose registry and management systems should allow fulfilling the regulatory requirements of the national and European regulations and should help to extract the basic information on exposure parameters for the practitioners and referrers.

It should be noted as a limitation of this initial step, that not all of the 11 members of the WG have been able to complete the survey and probably the opinion of the experts have not been formally contrasted with other colleagues in their respective countries or scientific or professional societies. This could be a second step, after an initial experience with the implementation of the Directive. This initial experience will allow detecting some practical difficulties from the approaches taken in the different European countries.

A working group on “Dose management” was launched recently by EuroSafe Imaging and the recommendations included in this paper will be considered therein.

## Supplementary information

**Additional file 1.** Questionnaire distributed to the members of the EuroSafe Imaging Working Group on “Dosimetry for imaging in clinical practice”.

## Data Availability

All data generated or analysed during this study are included in this published article.
