# Supplementary material for: Harmonisation of imaging dosimetry in clinical practice: practical approaches and guidance from the ESR EuroSafe Imaging initiative
Source: Insights Imaging. 2020 Mar 30;11:54. doi: 10.1186/s13244-020-00859-6 (PMC7105556; doi:10.1186/s13244-020-00859-6)
Supplement: Supplementary file 1 — Additional file 1. Questionnaire distributed to the members of the EuroSafe Imaging Working Group on “Dosimetry for imaging in clinical practice”. [file 13244_2020_859_MOESM1_ESM.pdf]

APPENDIX 1

Questionnaire distributed to the members of the EuroSafe Imaging Working Group on "Dosimetry for imaging in clinical practice"

1) **Information on patient exposure for patients** (**Art. 58.b of the European directive 2013/59/EURATOM**: *Member States shall ensure that information relating to patient exposure forms part of the report of the medical radiological procedure).* **This information should be:**

   a) Just the dose values and units, reported by the X-ray system. **Yes / No** / **don't know / don't have opinion**
   b) The estimation of the effective dose value (mSv) **Yes / No**/ **don't know / don't have opinion**
   c) The estimation of the equivalent time in background radiation **Yes / No**/ **don't know / don't have opinion**
   d) Other options (please, describe).

2) **Individual optimisation (Art. 5.b of the European directive**: *The optimisation of the protection of individuals subject to medical exposure shall apply to the magnitude of individual doses and be consistent with the medical purpose of the exposure).* **This should be made:**

   a) By comparing a group of procedures with Diagnostic Reference Levels (DRLs) **Yes / No**/ **don't know / don't have opinion**
   b) Considering individual optimisation if individual doses are much higher than DRLs **Yes / No**/ **don't know / don't have opinion**
   c) Considering patient and staff doses for interventional procedures **Yes / No**/ **don't know / don't have opinion**
   d) Other options (please, describe).

3) **Accidental and unintended exposures (Art. 63.c of the European directive***: Member States shall ensure that for all medical exposures the undertaking implements an appropriate system for the record keeping and analysis of events involving or potentially involving accidental or unintended medical exposures, commensurate with the radiological risk posed by the practice).* **The priorities should be:**

   a) If suspected an accidental or unintended exposure, record and analyse the dose parameters (based on physical quantities) and produce a report for the quality assurance committee. **Yes / No** / **don't know / don't have opinion**
   b) To produce an educational note for radiographers, radiologists and medical physicists. **Yes / No**/ **don't know / don't have opinion**
   c) Other options (please, describe).

4) **Dosimetric trigger levels** (for individual procedures) **should be established:**

   a) For interventional procedures to alert on the risk of potential skin injuries. **Yes /No**/ **don't know / don't have opinion**
   b) For all imaging procedures when dosimetric values are much higher than the DRLs. **Yes /No**/ **don't know / don't have opinion**

c) Other options (please, describe).

5) The **comparison with Diagnostic Reference Levels (DRLs) should be made:**

a) At least, once per year. **Yes /No**/ **don't know / don't have opinion**
b) Continuously if the patient dose registry allow to do it. **Yes /No**/ **don't know / don't have opinion**
c) When required by the competent authority. **Yes /No**/ **don't know / don't have opinion**
d) After changes in the X-ray unit or changes in the imaging protocols **Yes /No**/ **don't know / don't have opinion**
e) Other options (please, describe).

6) **The dose registry and management** systems should:

a) Include a validation/correction factor (periodically certified by a medical physics expert). **Yes /No**/ **don't know / don't have opinion**
b) Allow to fulfil the regulatory requirements (directive 2013/59/EURATOM) on patient dose registration. **Yes / No**/ **don't know / don't have opinion**
c) Help in the procedures optimisation (including individual optimisation) and alerting on accidental or unintended exposures. **Yes/ No**/ **don't know / don't have opinion**
d) Allow to manage the dosimetric quantities calculated or measured by the X-ray systems. **Yes / No**/ **don't know / don't have opinion**
e) Other options (please, describe).

7) The **practitioner should have information on:**

a) The physical quantities offered by the X-ray system for the different imaging modalities. **Yes / No**/ **don't know / don't have opinion**
b) The effective doses for each procedure. **Yes / No**/ **don't know / don't have opinion**
c) The diagnostic reference levels. **Yes /No**/ **don't know / don't have opinion**
d) Other options (please, describe).

8) The **referrer should have information on:**

a) The physical quantities offered by the X-ray system. **Yes / No**/ **don't know / don't have opinion**
b) The effective doses. **Yes / No**/ **don't know / don't have opinion**
c) The diagnostic reference levels. **Yes /No**/ **don't know / don't have opinion**
d) Other options (please, describe).
